# Preparation breeds success: Brain activity predicts remembering

**DOI:** 10.1016/j.cortex.2018.04.009

**Published:** 2018-09

**Authors:** Jane E. Herron, Lisa H. Evans

**Affiliations:** Cardiff University Brain Research Imaging Centre (CUBRIC), School of Psychology, Cardiff University, Cardiff, Wales, UK

**Keywords:** Episodic memory, Retrieval mode, Retrieval orientation, Event-related potentials (ERPs), Preparation

## Abstract

Successful retrieval of episodic information is thought to involve the adoption of memory states that ensure that stimulus events are treated as episodic memory cues (retrieval mode) and which can bias retrieval toward specific memory contents (retrieval orientation). The neural correlates of these memory states have been identified in many neuroimaging studies, yet critically there is no direct evidence that they facilitate retrieval success. We cued participants before each test item to prepare to complete an episodic (retrieve the encoding task performed on the item at study) or a non-episodic task. Our design allowed us to separate event-related potentials (ERPs) elicited by the preparatory episodic cue according to the accuracy of the subsequent memory judgment. We predicted that a correlate of retrieval orientation should be larger in magnitude preceding correct source judgments than that preceding source errors. This hypothesis was confirmed. Preparatory ERPs at bilateral frontal sites were significantly more positive-going when preceding correct source judgments than when preceding source errors or correct responses in a non-episodic baseline task. Furthermore this effect was not evident prior to recognized items associated with incorrect source judgments. This pattern of results indicates a direct contribution of retrieval orientation to the recovery of task-relevant information and highlights the value of separating preparatory neural activity at retrieval according to subsequent memory accuracy. Moreover, at a more general level this work demonstrates the important role of pre-stimulus processing in ecphory, which has remained largely neglected to date.

## 1 Introduction

Many of the stimuli that we encounter in everyday life have associations with the past. There are many people who we meet, places that we pass and items that we see or use that would be excellent cues for events from our personal past. For example, when I look at my watch to tell the time I could recover details of the episode when I was given it as a gift. However these memories typically do not come to mind. Given the abundance of cues that we are confronted with it is perhaps surprising that we are not constantly reminiscing. What this demonstrates is that having a cue and a relevant past experience does not guarantee the recovery of information. So what else is required? According to Tulving (1983), in order for an individual to remember a particular episode they need to enter a cognitive state where stimulus events are treated as episodic memory cues. Thus another important prerequisite for remembering is that the individual should be in a state of mind that is focused on their personal past, known as retrieval mode.

We know little about retrieval mode. The work that has been conducted in this area has tended to use neuroimaging techniques, due to the difficulty in studying cognitive states using behavioral measures alone. In order to reveal neural indices of retrieval mode, paradigms have been used where participants switch between different classes of tasks, episodic versus non-episodic, as retrieval mode should only be engaged when individuals are required to retrieve episodic information (Rugg & Wilding, 2000). One of the first studies in this area (Duzel et al., 1999) recorded direct current event-related potentials (ERPs) while participants switched between completing separate blocks of a recognition memory task and a semantic judgment task. A cue, which indicated which task the participant should complete, was presented for 2 s prior to the first of four test words. ERPs associated with the episodic retrieval cue were more positive-going compared to the semantic cue, with differences emerging just after the presentation of the task cue and being sustained for the rest of the block. This effect was maximal at a right frontopolar site, which is consistent with findings from hemodynamic studies of retrieval mode where activation in the right prefrontal cortex has been found (Duzel et al., 2001, Lepage et al., 2000, Nyberg et al., 1995, Velanova et al., 2003).

More recent studies using ERPs have presented the task cue before each test item and asked participants to switch between completing tasks with different retrieval demands. This gives an interval, of around 2 s, where the participant knows the task they will need to complete on the subsequent test item. During this preparatory period retrieval mode would be anticipated to be engaged, and importantly neural activity is not contaminated by indices of memory retrieval. Several studies have found differences in slow wave activity at frontal scalp locations for cues indicating preparation for an episodic versus a non-episodic task and this pattern of data has been interpreted as the electrophysiological signature of retrieval mode (Evans et al., 2015, Herron and Wilding, 2004, Herron and Wilding, 2006a, Morcom and Rugg, 2002, Wilckens et al., 2011). The divergences can onset quite early (e.g. 300 ms; Herron & Wilding, 2004) but commonly start around 800 ms from the onset of the preparatory cue and are sustained until the test item is presented. In the majority of studies, this effect has been observed on the second trial of the episodic task rather than the first. Drawing from the task-switching literature (Monsell, 2003), it has been concluded on the basis of these findings that retrieval mode cannot be successfully initiated until at least one trial of the episodic task has been completed (Herron and Wilding, 2004, Herron and Wilding, 2006a, Morcom and Rugg, 2002), a phenomenon referred to by Duzel et al. (2001) as ‘neurocognitive inertia’. More recently, however, Evans et al. (2015) demonstrated that ERP correlates of retrieval mode can in fact be obtained on the first trial of the episodic task if the contents of the episodic and non-episodic tasks are equated (in this instance, remember the test probe's prior location or make perceptual location judgments) and/or the trial sequence is predicable, thus reducing the cognitive load required to switch between tasks.

Whereas retrieval mode is a general episodic memory state initiated whenever episodic retrieval is required and which remains invariant across different retrieval goals, content-specific memory states – termed ‘retrieval orientations’ – are engaged when there is a requirement to retrieve specific kinds of episodic information (Rugg & Wilding, 2000). Retrieval orientations are thought to influence stimulus processing to facilitate the retrieval of task-relevant information, and a wide variety of ERP and fMRI studies have therefore contrasted stimulus-locked neural activity across memory tasks with varying retrieval goals to obtain their neural correlates (e.g. Hornberger et al., 2006a, Hornberger et al., 2006b, Bridger et al., 2009, Dzulkifli and Wilding, 2005, Herron and Rugg, 2003, Hornberger et al., 2004, Johnson and McGhee, 2015, McDuff et al., 2009, Morcom and Rugg, 2012, Rosburg et al., 2013, Rosburg et al., 2014, Werkle-Bergner et al., 2005, Woodruff et al., 2006). In keeping with its definition, neural correlates of retrieval orientation vary according to specific retrieval goals.

Preparatory correlates of retrieval orientation have also been studied in ERP experiments which cue participants to switch between different episodic memory tasks (Herron and Wilding, 2004, Herron and Wilding, 2006b). Cues directing participants to prepare to retrieve either location-based information or encoding task elicited differential slow–wave activity at left anterior electrode sites during the cue-stimulus interval. Unlike the majority of studies examining mode, this preparatory correlate of retrieval orientation was observed on the first trial of the task when two episodic cue-types were employed (Herron & Wilding, 2006b). An fMRI study which similarly cued participants to retrieve either encoding list or encoding task reported activation in left lateral anterior prefrontal cortex (Simons, Gilbert, Owen, Fletcher, & Burgess, 2005). The observation that this activation peaked 4s prior to recollection and was additionally evident on trials containing no retrieval stimuli led the authors to propose that this region may have given rise to the preparatory ERP effect reported by Herron and Wilding (2004).

While there is now a substantial body of evidence supporting the existence of retrieval mode and orientation, a critical issue that has not yet been resolved is the relationship between the adoption of these memory states and success in recovering episodic information from memory. If they ensure that stimulus events are treated as episodic memory cues then their engagement should lead to enhanced episodic memory. In the case of retrieval mode, its engagement would be predicted to facilitate episodic memory of any kind, benefiting recognition and both noncriterial (i.e. recollection of details that are irrelevant to task demands, Yonelinas & Jacoby, 1996) and criterial recollection. The initiation of retrieval orientations should lead to more selective improvements in source accuracy (criterial recollection) by facilitating the retrieval of task-relevant contextual information. Existing evidence is mixed, with one study reporting improvements in source accuracy (but not recognition accuracy) on trials following neural evidence of retrieval mode initiation (Herron & Wilding, 2006a) and others failing to detect improvements in recognition or source accuracy following neural evidence of either mode or orientation (Herron and Wilding, 2004, Herron and Wilding, 2006b, Morcom and Rugg, 2002, Wilckens et al., 2011). Importantly, these studies were not designed to directly address this question, as preparatory neural activity was not separated according to retrieval success or failure. Moreover, this would not have been possible to complete in these studies as accuracy levels were high and there would have been insufficient trial numbers to form an average to memory errors. Therefore previous studies have only been able to note the correspondence, or not, between the presence of the neural index of mode or orientation and an improvement in memory accuracy.

The current study was designed to experimentally assess this issue by separating ERPs associated with preparatory episodic cues according to the accuracy of the subsequent memory judgment in a source memory task. This is an approach which has been used widely in the encoding phase of experiments, where neural activity is sorted according to whether the participant subsequently remembers or forgets the item at test, and has provided influential insights into memory (the ‘subsequent memory effect’; Paller et al., 1987, Paller and Wagner, 2002). Here we adopt the same logic but applied to the preparatory period of the retrieval phase. If preparatory neural activity linked to memory states facilitates episodic retrieval it would be anticipated that this electrophysiological index will be significantly larger in magnitude when preceding accurate memory judgments than when preceding memory errors. Furthermore, it will be of interest to determine whether this index predicts recognition success independent of criterial source accuracy (indicative of mode) or whether it differentiates recognized items associated with correct and incorrect source judgments (indicative of orientation). While previous studies have obtained neural correlates of retrieval orientation by contrasting neural activity in two (or more) tasks that vary in their episodic requirements – thereby contrasting different orientations – it is also theoretically possible to examine the extent to which a single orientation is engaged by conditionalising preparatory neural activity within a task according to criterial source accuracy.

## 2 Materials and methods

### 2.1 Participants

Twenty-nine healthy participants gave informed consent before the experiment and received a monetary reward for participating. Two were excluded from analysis because performance on the source memory judgment was at chance and a further three were excluded because they failed to contribute at least 16 artifact-free ERP trials to each of the experimental conditions of interest. All remaining 24 participants were right-handed native English speakers and 20 were female (mean age: 21 years, range: 18–26). Ethical approval for the study was granted by Cardiff University's School of Psychology ethics committee. Data underpinning this publication is available on request.

Previous ERP studies which have examined retrieval mode have effect sizes ranging from .47 to .69 (Cohen's d_z_; Duzel et al., 1999, Evans et al., 2015, Herron and Wilding, 2004, Herron and Wilding, 2006a, Morcom and Rugg, 2002). Assuming a power level of .80 and an alpha of .05 this leads to an estimated average sample size of 22 to obtain similar effects. A sample size of 24 participants was predetermined based on these a priori power analyses and counterbalancing constraints.

### 2.2 Design

Stimuli were 480 nouns (concreteness range = 500–700) selected from the MRC psycholinguistic database (Coltheart, 1981) with Kucera-Francis frequencies of 1–9 per million. Words were 3–9 letters long and were presented at central fixation in white capitalized Times New Roman font on a black background. The experiment consisted of a practice block and then five study-test blocks. At study participants alternated between an animate/inanimate task and a pleasant/unpleasant task four times within each block, performing the specified encoding task until the alternate study instructions appeared. Each study list comprised 72 words with an additional 24 new words at test. At test each word was preceded by one of two cues (X or O) which directed participants to prepare to complete either the episodic or the non-episodic task (syllable counting).

Each test block contained 64 episodic cues and 32 syllable cues. Each cue type was presented for 2 consecutive trials to permit data associated with each cue type to be separated according to whether the cue was different from that on the preceding trial (switch trials) or the same (stay trials). This was because ERP correlates of retrieval mode have frequently shown a delayed onset, being observed only on ‘stay’ trials in previous studies (e.g. Herron and Wilding, 2004, Herron and Wilding, 2006a, Morcom and Rugg, 2002, Wilckens et al., 2011). In total there were 320 episodic cues: 240 preceded an old word and 80 a new word, split equally between switch and stay trials. There were 160 non-episodic (syllable) cues, resulting in 80 switch trials and 80 stay trials (120 cues preceding an old word and 40 before a new word: this factor was collapsed for the non-episodic cues). An additional 16 filler trials were also inserted into each block. These followed the same structure as all the other trials but were always single trials of the non-episodic task to separate the greater number of episodic trial pairs. These trials were not included in analyses. The old/new status of words, the mapping of X/O to task and the assignment of words to the episodic or syllable task were fully counterbalanced across participants.

### 2.3 Procedure

At study, participants performed the encoding task specified by the onscreen instruction, responding via button press with their left or right hand. A fixation asterisk (1000 ms) preceded the study word (300 ms) then the screen remained black until 500 ms after a response was made.

At test, episodic cues required participants to judge whether the subsequent word had been presented in the animate/inanimate task, the pleasant/unpleasant task, or was new. Non-episodic cues required participants to judge whether the word consisted of one, two, or more than two syllables. Participants responded via button press, using the index finger of one hand for new/one syllable responses and the index and middle fingers of the other hand for the remaining responses, and were encouraged to balance speed and accuracy equally. The preparatory cue (300 ms) was followed by an asterisk (2000 ms) and the test word (300 ms) then the screen remained black until 500 ms after a response was made. Participants were instructed to maintain fixation at the centre of the screen throughout each test phase.

### 2.4 Electroencephalogram (EEG) acquisition and analysis

EEG was recorded with a Biosemi Active Two amplifier from 32 locations based on the International 10–20 system (Jasper, 1958). Additional electrodes were placed on the mastoid processes. EOG was recorded from above and below the left eye (VEOG) and from the outer canthi (HEOG). EEG (range DC-419 Hz; sampling rate 2048 Hz) was acquired referenced to linked electrodes located midway between POz and PO3/PO4 respectively, and was re-referenced off-line to linked mastoids. Trials containing HEOG artifact and non-blink-related EOG artifact were rejected, as were trials containing A/D saturation or baseline drift exceeding ±80 μV. This was completed by applying an automated algorithm for detecting artifacts and blinks followed by visual inspection of individual trial data to ensure that all trials containing artifact had been excluded and blinks detected. The experimenter was blind to trial type during this process. EOG blink artifacts were corrected using a linear regression estimate (Semlitsch, Anderer, Schuster, & Presslich, 1986) in line with previous studies of retrieval mode (Evans et al., 2015, Herron and Wilding, 2004, Herron and Wilding, 2006a, Morcom and Rugg, 2002). Data from each participant were then visually compared pre- and post-correction to ensure that blink correction had been successful. A 7-point binomially weighted smoothing filter was applied prior to analysis. Data was filtered off-line (.03–40 Hz) and downsampled to 125 Hz, resulting in a total epoch length of 2048 ms with a 104 ms baseline relative to which all mean amplitudes were computed.

Averaged ERPs were formed for each participant to the episodic cues separated according to whether source memory judgments were correct (*Episodic Hit-Hits*) or not (*Episodic Errors*). The latter category consisted of a weighted average of recognized items associated with incorrect source judgments (*Episodic Hit-Misses)* and items that were not recognized at all (*Episodic Miss-Misses)*. These were contrasted with ERPs elicited by the non-episodic cues preceding correct responses (*Non-Episodic Hits*). The ERP trial numbers contributing to these averages were as follows: Episodic Hit-Hits (Switch) = 78 (46–102), Episodic Errors (Switch) = 37 (17–72), Non-Episodic Hits (Switch) = 68 (45–76), Episodic Hit-Hits (Stay) = 82 (53–102), Episodic Errors (Stay) = 34 (16–65), Non-Episodic Hits (Stay) = 67 (39–77). Prior research has identified effects of cue type at frontal sites from 800 ms onwards (Evans et al., 2015, Herron and Wilding, 2004, Herron and Wilding, 2006a, Wilckens et al., 2011), therefore mean amplitudes of averaged ERPs time-locked to cues were calculated for the 800–1900 ms latency region at 10 anterior electrode sites (F7/F8, F5/F6, F3/F4, F1/F2, Fp1/Fp2).

A further analysis was conducted on a subgroup of 16 participants who made enough Episodic Hit-Miss responses to allow ERPs to be formed for this response category. To reiterate, an Episodic Hit-Miss refers to a correctly recognized studied item associated with an incorrect source judgment. ERP trial numbers for this response category were: Switch = 26 (16–51), Stay = 25 (16–50).^1^ This analysis was analogous to that described above, with Episodic Hit-Hits, Episodic Hit-Misses, and Non-Episodic Hits measured between 800 and 1900 ms at the same 10 anterior electrode sites. The purpose of this analysis was to constrain functional interpretations of accuracy effects obtained in the primary analysis. As retrieval orientations are thought to facilitate criterial recollection of task-relevant contextual information in accordance with retrieval goals, a preparatory correlate of retrieval orientation should differentiate Episodic Hit-Hits from Episodic Hit-Misses, with no differentiation between Non-Episodic Hits and Episodic Hit-Misses. Conversely, retrieval mode is assumed to remain invariant across episodic recognition and source memory requirements, with preparatory ERP correlates of retrieval mode evident during item recognition (Duzel et al., 1999, Morcom and Rugg, 2002) and different source memory tasks (Herron and Wilding, 2004, Herron and Wilding, 2006a). It follows from this that a correlate of mode should predict the ability to correctly recognize a studied item independent of criterial source accuracy, and that ERPs preceding Episodic Hit-Hits and Episodic Hit-Misses should therefore not differ but should both diverge from ERPs preceding Non-Episodic Hits.

All ANOVA analyses included the Greenhouse-Geisser correction for non-sphericity where necessary (Greenhouse & Geisser, 1959). Epsilon-corrected degrees of freedom are given in the text. A significance level of *p* < .05 was adopted, unless otherwise stated. Main effects and highest order interactions obtained are reported below. Within-subjects confidence intervals have been calculated according to the procedure of Loftus and Masson (1994).

## 3 Results

### 3.1 Behavior

Behavioral test data separated according to whether responses were made on switch or stay trials are shown in Table 1. Repeated measures ANOVA of Episodic Hit-Hits and Non-Episodic Hits including factors of Task (episodic, non-episodic) and Trial Type (switch, stay) revealed a main effect of Task (*F*_(1,23)_ = 36.20, *p* < .001) and a Task x Trial Type interaction (*F*_(1,23)_ = 4.75, *p* = .040). This reflected an increase in accuracy from switch (*M* = .68, 95% CI = [.63, .73]) to stay trials (*M* = .71, 95% CI = [.67, .76]) in the episodic task only (*t*_(1,23)_ = 2.86, *p* = .009, Cohen's d_z_ = .58, Hedges g_av_ = .22). In order to determine the loci of this improvement in accuracy t-tests between trial types were conducted on both old/new discrimination (p_hit_–p_false alarm_) and source accuracy conditionalised on correct recognition (i.e. study items attracting correct source judgments expressed as a proportion of correctly recognized items). Old/new discrimination was significantly higher on stay (M = .78, 95% CI = [.74, .82]) than on switch (*M* = .71, 95% CI = [.66, .76]) trials (*t*_(23)_ = 3.01, *p* = .006, Cohen's d_z_ = .61, Hedges g_av_ = .51). Conditional source accuracy was also significantly higher on stay (M = .79, 95% CI = [.75, .83]) than on switch trials (*M* = .77, 95% CI = [.73, .81]) *t*_(23)_ = 2.23, *p* = .036, Cohen's d_z_ = .45, Hedges g_av_ = .20). A t-test performed on measures of response criterion (Br) on switch (*M* = .51, 95% CI = [.4 to .62]) and on stay trials (*M* = .44, 95% CI = [.34 to .54]) did not find any effect of trial sequence on this measure (t_*(23)*_ = 1.20, *p* = .24, Cohen's d_z_ = .24, Hedges g_av_ = .28).

**Table 1:** Mean response accuracy and associated reaction times (in ms) for the episodic and non-episodic tasks on switch and stay trials (standard deviations in parentheses).

	Switch Trials	Stay Trials
**Accuracy**		
Episodic Hit-Hits	.68 (.12)	.71 (.11)
Episodic Hit-Misses	.20 (.09)	.18 (.08)
Episodic Miss-Misses	.12 (.07)	.11 (.06)
Correct Rejections	.84 (.13)	.88 (.10)
Non-Episodic Hits	.88 (.09)	.87 (.11)
**Reaction Times**		
Episodic Hit-Hits	1999 (444)	1751 (360)
Episodic Hit-Misses	2132 (546)	1959 (534)
Episodic Miss-Misses	1849 (584)	1709 (384)
Correct Rejections	1550 (388)	1414 (338)
Non-Episodic Hits	1320 (514)	1254 (431)

ANOVA of RT data associated with Episodic Hit-Hits and Non-Episodic Hits on switch and stay trials revealed a Task x Trial Type interaction (*F*_(1,23)_ = 22.80, *p* < .001) as well as a main effect of Task (*F*_(1,23)_ = 54.07, *p* < .001) and Trial Type (*F*_(1,23)_ = 32.68, *p* < .001). The interaction reflected the fact that RTs decreased significantly from switch (*M* = 1699 ms, 95% CI = [1519, 1879]) to stay trials (*M* = 1451 ms, 95% CI = [1311, 1591]) for Episodic Hit-Hits (t_*(23)*_ = 8.17, *p* < .001, Cohen's d_z_ = 1.67, Hedges g_av_ = .61) but not for Non-Episodic Hits (t_*(23)*_ = 1.81, *p* = .084, Cohen's d_z_ = .37, Hedges g_av_ = .14).

### 3.2 ERP analyses

ANOVA of mean amplitudes taken from the ERPs elicited by cues at frontal sites included the factors of Response Type (Episodic Hit-Hits, Episodic Errors, Non-Episodic Hits), Trial Type (switch, stay), Hemisphere (left, right) and Site. The Site factor had five levels: inferior (F7/F8), midlateral (F5/F6), superior (F3/F4), midline (F1/F2) frontopolar (Fp1/Fp2) sites. There was a main effect of Response Type (*F*_(2.0,45.3)_ = 5.17, *p* = .010) and a significant interaction between Response Type, Trial Type and Site (*F*_(4.4,100.4)_ = 4.36, *p* = .002). In light of the ERP differences reported previously according to trial-type (Evans et al., 2015, Herron and Wilding, 2004, Herron and Wilding, 2006a), this interaction was followed up by examining switch and stay trial data separately.

**Fig. 1:**
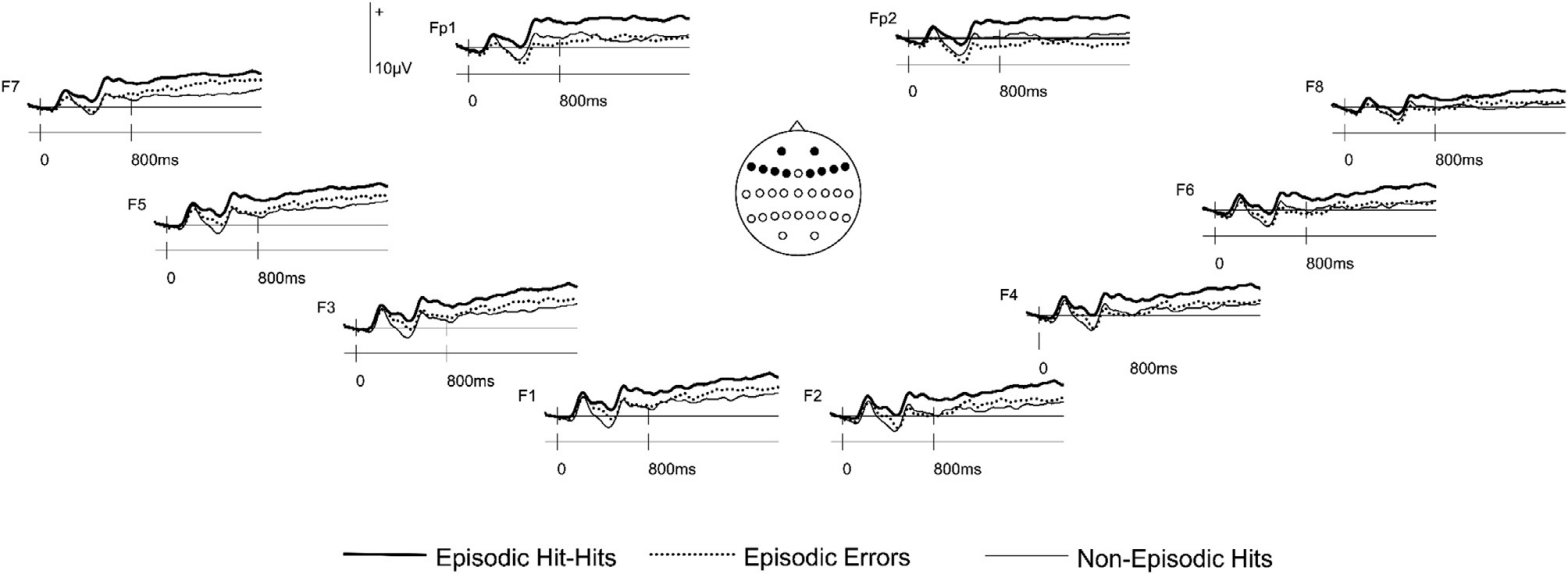
Grand average ERP waveforms (N = 24) time-locked to switch trial cues preceding Episodic Hit-Hits, Episodic Errors and Non-Episodic Hits at the 10 anterior sites analyzed.

**Fig. 2:**
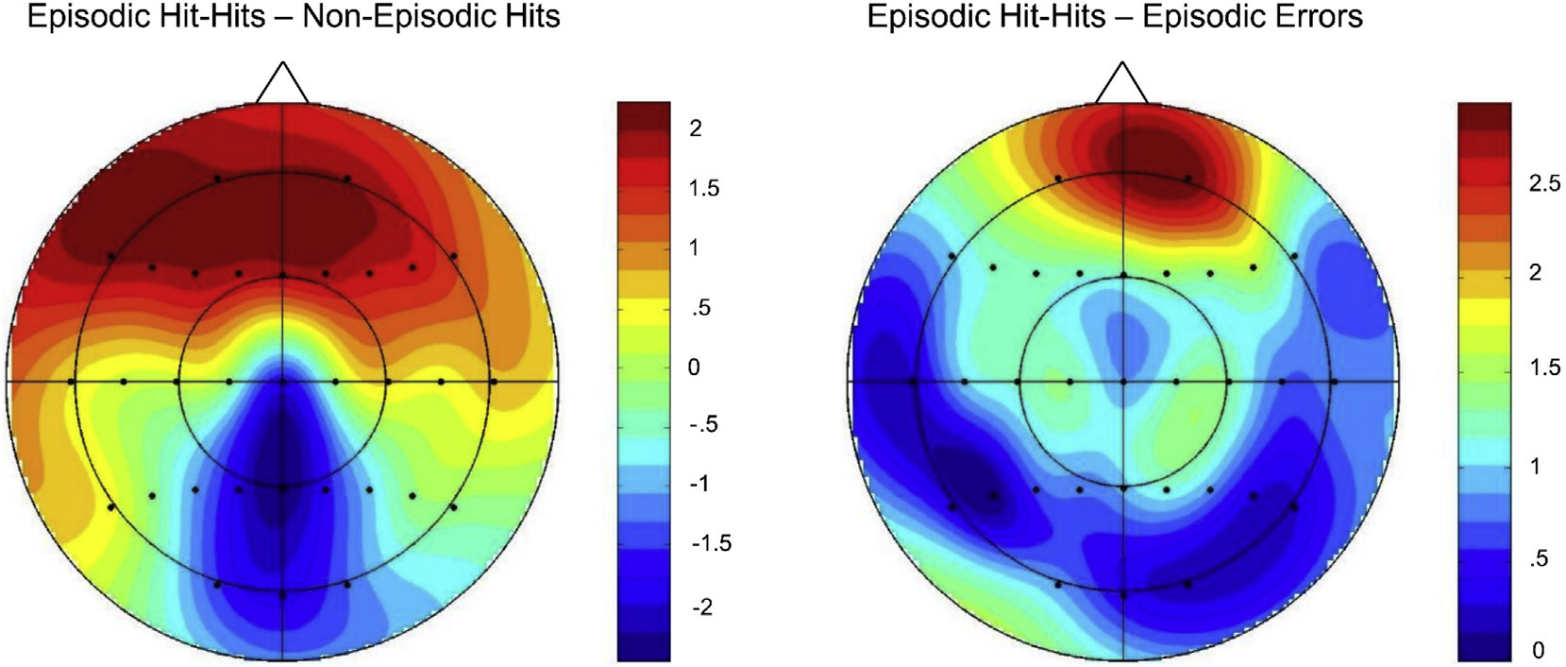
Topographic maps showing the scalp distributions of cue-related ERP effects on switch trials between 800 and 1900 ms. Data were formed by subtracting averaged ERP amplitudes associated with the response conditions indicated above each scalp map. Each map is scaled proportionately between the minimum and maximum values denoted. The maps were computed using a spherical spline interpolation (Perrin, Pernier, Bertrand, & Echallier, 1989).

Analysis of ERPs on switch trials, see Fig. 1, revealed a main effect of Response Type (*F*_(1.9,43.3)_ = 12.83, *p* < .001) and an interaction between Response Type and Site (*F*_(4.3,97.8)_ = 3.52, *p* = .008). Pairwise comparisons were then completed between the three response types, each comparison being between pairs of response types and incorporating the original factors of Response Type, Hemisphere and Site. There was a significant main effect of Response Type between Episodic Hit-Hits and Non-Episodic Hits (*F*_(1,23)_ = 17.99, *p* < .001). Moreover, a main effect of Response Type was observed between Episodic Hit-Hits and Episodic Errors (*F*_(1,23)_ = 17.66, *p* < .001), which was moderated by an interaction with Site (*F*_(2.2,50.6)_ = 6.57, *p* = .002). Post-hoc analyses conducted at each of the 5 Site levels (corrected alpha level = .01), revealed differences at Fp1/Fp2 (*F*_(1,23)_ = 27.79, *p* < .001), F1/F2 (*F*_(1,23)_ = 9.14, *p* = .006), F3/F4 (*F*_(1,23)_ = 10.82, *p* = .003) and F5/F6 (*F*_(1,23)_ = 15.95, *p* = .001) locations, but not at F7/F8 (*F*_(1,23)_ = 6.82, *p* = .016). These outcomes are due to a greater relative positivity for Episodic Hit-Hits compared to Episodic Errors at bilateral frontal locations (see Fig. 1, Fig. 2). Finally, for the contrast between Episodic Errors and Non-Episodic Hits there was an interaction between Response Type and Site (*F*_(2.4,55.4)_ = 4.02, *p* = .018). This appears to be due to relatively greater positivity for Episodic Errors at F7/F8 and greater negativity at frontopolar sites. However in follow-up post-hoc tests effects of Response Type were not reliable at either F7/F8 (*F*_(1,23)_ = 2.88, *p* = .10) or frontopolar sites (*F*_(1,23)_ = 1.10, *p* > .250).

Analysis of ERPs associated with the three response types on stay trials revealed an interaction between Response Type and Hemisphere (*F*_(1.9,43.6)_ = 4.17, *p* = .024). No reliable effect of Response Type was revealed in the contrast between Episodic Hit-Hits and Episodic Errors (*F*_(1,23)_ = .59, *p* > .250) or between Episodic Errors and Non-Episodic Hits (*F*_(1,23)_ = 3.08, *p* = .093). A pairwise comparison of Episodic Hit-Hits and Non-Episodic Hits revealed a Response Type x Hemisphere crossover interaction (*F*_(1,23)_ = 6.83, *p* = .016) reflecting relatively greater negativity for Episodic Hit-Hits at left anterior sites and a smaller effect of opposite polarity at right hemisphere sites. However the effects of Response Type were not significant in post-hoc tests (corrected alpha level = .025) conducted separately at left (*F*_(1,23)_ = 1.62, *p* = .216) and right hemisphere sites (*F*_(1,23)_ = .02, *p* = .892).

These outcomes indicate the sensitivity of preparatory ERPs to retrieval success. In order to investigate the functional significance of this sensitivity with greater precision, further analyses were performed on a subgroup of 16 participants for whom ERPs could be formed for Episodic Hit-Misses. These analyses employed the same structure as the whole group analyses, with the exception that the factor of Response Type incorporated Episodic Hit-Hits, Episodic Hit-Misses and Non-Episodic Hits. In the global ANOVA there was a main effect of Response Type (*F*_(1.6,24.3)_ = 5.44, *p* = .016), as well as a significant interaction between Response Type, Trial Type and Site (*F*_(3.9,59.0)_ = 3.80, *p* = .008).

**Fig. 3:**
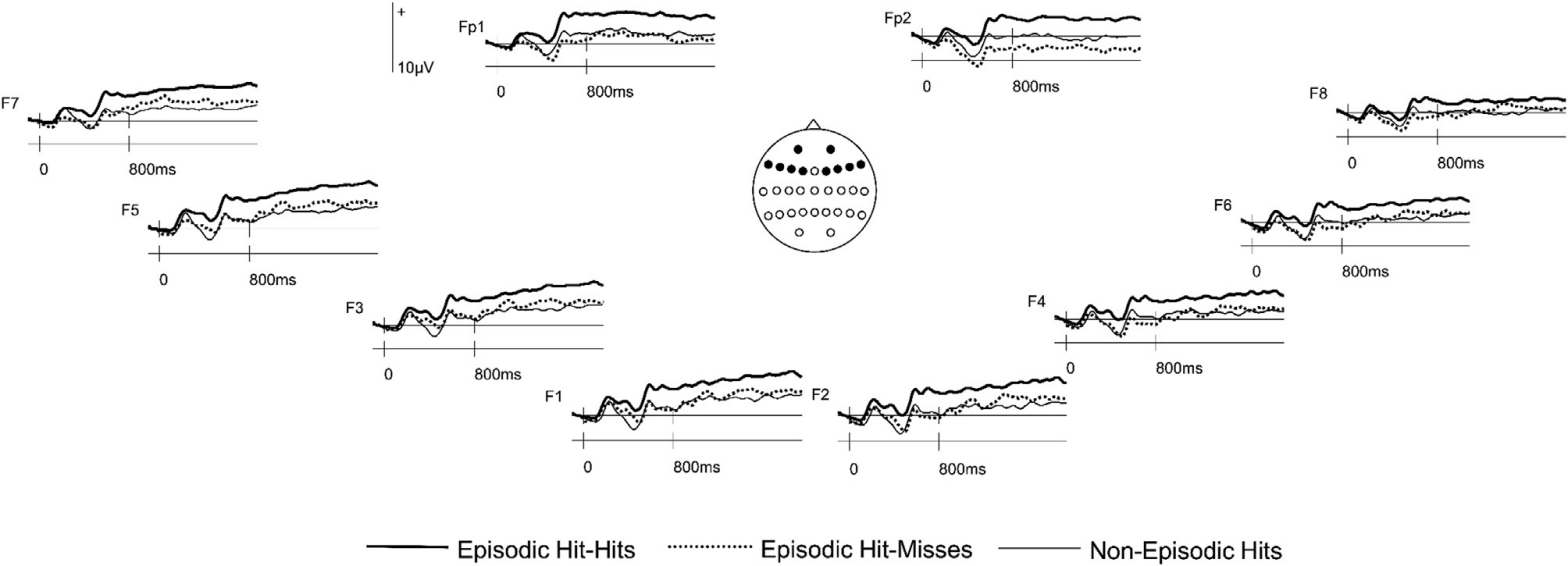
Grand average ERP waveforms (N = 16) time-locked to switch trial cues preceding Episodic Hit-Hits, Episodic Hit-Misses and Non-Episodic Hits at the 10 anterior sites analyzed.

In follow-up analyses reliable outcomes occurred on switch trials only (see Fig. 3). Here there was a main effect of Response Type (*F*_(1.8,27.1)_ = 10.88, *p* < .001) and an interaction between Response Type and Site (*F*_(3.8,57.0)_ = 3.10, *p* = .024). A pairwise comparison between Episodic Hit-Hits and Episodic Hit-Misses revealed a main effect of Response Type (*F*_(1,15)_ = 13.91, *p* = .002) and an interaction between Response Type and Site (*F*_(1.8,27.5)_ = 5.51, *p* = .011). Post-hoc analyses conducted at each of the 5 site locations (corrected alpha level = .01) revealed differences at Fp1/Fp2 (*F*_(1,15)_ = 13.31, *p* = .002), F1/F2 (*F*_(1,15)_ = 12.67, *p* = .003), F3/F4 (*F*_(1,15)_ = 14.87, *p* = .002) and F5/F6 (*F*_(1,15)_ = 14.19, *p* = .002) locations, but not at F7/F8 (*F*_(1,15)_ = 6.19, *p* = .025). These outcomes reflect a greater relative positivity for Episodic Hit-Hits compared to Episodic Hit-Misses at bilateral frontal locations. This is the same pattern of results that was found in the full sample of participants when contrasting Episodic Hit-Hits with Episodic-Errors.

There was also a main effect of Response Type in the comparison between the ERPs elicited by Episodic Hit-Hits and Non-Episodic Hits (*F*_(1,15)_ = 14.18, *p* = .002). There was no significant main effect of Response Type in the pairwise comparison of Episodic Hit-Misses and Non-Episodic Hits (*F*_(1,15)_ = .12, *p* > .250). A crossover interaction between Response Type and Site (*F*_(2.1,31.9)_ = 3.79, *p* = .031) reflected greater positivity for Episodic Hit-Misses maximal at inferior frontal sites (F7/F8) and greater positivity for Non-Episodic Hits at frontopolar sites. However, neither the effect at inferior frontal (*F*_(1,15)_ = 1.45, *p* = .248) nor frontopolar sites (*F*_(1,15)_ = 1.68, *p* = .215) was significant.

**Fig. 4:**
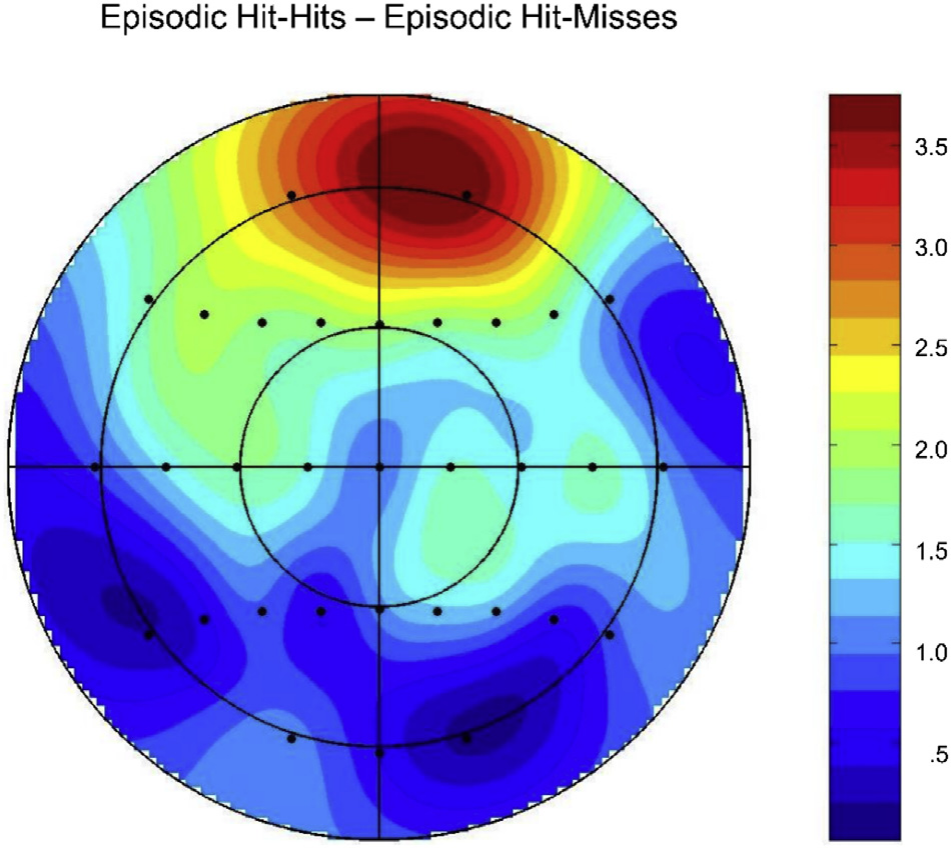
Topographic map showing the scalp distribution of the Episodic Hit-Hits condition minus Episodic Hit-Misses on switch trials between 800 and 1900 ms. The map is scaled proportionately between the minimum and maximum values denoted.

A topographic analysis compared the scalp distributions of the Episodic Hit-Hits minus Episodic Hit-Misses effect and the Episodic Hit-Hits minus Episodic Errors effects in the same subset of 16 participants reported here. This analysis was conducted on difference scores obtained by subtracting mean amplitudes (between 800 and 1900 ms) of each type of error-related ERP from the Episodic Hit-Hits ERPs, and the data were rescaled to avoid confounding changes in amplitude with changes in the shape of scalp distributions (McCarthy & Wood, 1985). The ANOVA incorporated the factors of Condition (Episodic Hits–Hits minus Episodic Errors, and Episodic Hits–Hits minus Episodic Hit-Misses) and Electrode Site (all 32 scalp electrode sites). No significant differences were observed between the two scalp distributions (*F*_(4.2,62.5)_ = 1.67, *p* = .17), indicating that the two effects were generated by the same neural populations (see Fig. 2, Fig. 4).

## 4 Discussion

The novel question addressed in this study is whether preparatory neural activity linked to the initiation of episodic memory states leads to success in recovering episodic information. Consistent with the outcomes of previous studies we found relatively more positive-going ERPs elicited by preparatory episodic memory cues compared to non-episodic cues at frontal scalp locations (Evans et al., 2015, Herron and Wilding, 2004, Herron and Wilding, 2006a, Morcom and Rugg, 2002, Wilckens et al., 2011). Crucially, this neural index predicted the accuracy of memory judgments. ERPs associated with episodic cues preceding retrieval success were significantly more positive-going than those preceding retrieval errors. Moreover, in a further analysis on a subset of 16 participants we investigated whether the index predicted criterial recollection of contextual information from the study phase or the more global ability to discriminate between studied and unstudied material. The former would support a role in retrieval orientation whereas the latter would support a role in retrieval mode. ERPs associated with accurate source memory judgments (Episodic Hit-Hits) diverged from those associated with recognized items attracting incorrect source judgments (Episodic Hit-Misses) and those associated with the non-episodic task at frontal sites. These outcomes are consistent with a direct contribution of retrieval orientation to the accuracy of source memory judgments.

The scalp distribution and the locus of the effect in the trial sequence provide further evidence linking it to orientation as opposed to mode. First, the effect was evident on switch trials rather than stay trials. ERP correlates of retrieval orientation were evident on switch trials in two previous experiments (Herron & Wilding, 2006b, Experiments 1a & 1b), whereas electrophysiological correlates of retrieval mode have been predominantly observed on stay trials (Duzel et al., 2001, Duzel et al., 1999, Herron and Wilding, 2004, Herron and Wilding, 2006a, Morcom and Rugg, 2002, Wilckens et al., 2011). The exception to this is a study by Evans et al. (2015), where the index of mode was observed on switch trials. The reasons suggested were the similarity in contents of the episodic and non-episodic tasks and/or the predicable trial sequence. However, neither of these factors apply to the current study. The absence of the effect on stay trials in the present study is consistent with the notion that it indexes task configuration processes that are involved in the initiation of orientation as opposed to its ongoing maintenance across items. There is some evidence that processes involved in the initiation and maintenance of retrieval orientations have dissociable ERP correlates (Herron and Wilding, 2006b, Herron et al., 2016), with those associated with maintenance predominantly being evident when retrieval requirements are blocked as opposed to alternating (Herron and Wilding, 2006b, Herron, 2018, Johnson and Rugg, 2006, Werkle-Bergner et al., 2005, Wilding and Nobre, 2001; but see; Herron et al., 2016). Second, the preparatory ERP effect observed here was broadly distributed across frontal sites. There was no statistical evidence for the right-sided lateralization consistently observed in studies of retrieval mode (Duzel et al., 2001, Duzel et al., 1999, Evans et al., 2015, Herron and Wilding, 2004, Herron and Wilding, 2006a, Morcom and Rugg, 2002). Three studies of preparatory ERP correlates of retrieval orientation have reported effects at left frontal sites during the time window analyzed here (Herron and Wilding, 2004, Herron and Wilding, 2006b, Experiments 1a & 1b), although earlier effects have also been observed at right posterior sites in a somewhat different experimental design (Herron et al., 2016), emphasizing the variable nature of retrieval orientations.

While it is theoretically possible that both mode and orientation could contribute to the preparatory effect observed here, the finding that the effect dissociates Episodic Hit-Hits from Episodic Hit-Misses (i.e. criterial recollection), is evident on switch trials, and showed no right-sided lateralization all converge to indicate a key role for orientation in this particular experiment. This raises fascinating new possibilities for the study of neural activity associated with retrieval orientations. Thus far, neuroimaging studies of retrieval orientation (both ERP and fMRI) have contrasted neural activity associated with different retrieval goals irrespective of memory success. In using this approach, researchers have contrasted neural activity associated with two (or more) retrieval orientations engaged during different episodic tasks. This contrast is an ambiguous one, as differences between correlates of distinct retrieval orientations could either be reflecting differential engagement of the same neural population, or – as seems more likely – activity in different content-specific brain regions or networks. Separating preparatory neural activity in a single source memory task according to subsequent criterial recollection (success or failure) allows neural activity associated with a single goal-directed orientation to be identified with far greater precision. Given the novelty of our approach and our findings, it will be important in future research both to replicate this and to extend it to other retrieval goals.

A key question that arises from our findings is: how does preparatory retrieval processing facilitate the accuracy of memory judgments? There are various ways in which it may do this, which are not mutually exclusive. One possibility is that it may enhance the quality and/or amount of episodic information revived by a studied item during retrieval. Some support for this account comes from a recent paper by Küper (2018) who examined episodic memory for perceptual and conceptual matches to studied items. Although this paper focused on retrieval mode, the author examined the influence of task-switching on neural correlates of recollection, linking these to preparatory neural activity. This was examined in two experiments: one which required participants to switch frequently between an episodic (recognition) and a non-episodic task and another where they completed a blocked episodic task. It was assumed that participants would not be able to initiate retrieval mode during task switching (and indeed no preparatory ERP indices of mode were detected here) but they would in the blocked design. The left-parietal old/new effect ERP, which has been regarded as the neural signature of recollection, was observed for conceptual matches in the blocked design only, leading to the proposal that retrieval mode may play an important role in the recollection of conceptual stimulus information (Küper, 2018). While these findings were presented within the framework of mode, it is plausible that retrieval orientations may similarly influence criterial recollection, and indeed task-specific orientation may potentially have contributed to performance on this task. While we were not able to make this kind of contrast here (having no blocked retrieval task), we observed a significant improvement in retrieval accuracy between switch and stay trials. As ERP evidence for the initiation of retrieval orientation was obtained on switch trials, and it is assumed that orientations are then maintained throughout subsequent trials in the same task as evidenced by paradigms using blocked designs; this finding is consistent with the hypothesis that maintaining an appropriate orientation enhances the availability of task-relevant episodic information.

A second possibility is that episodic memory states may facilitate the accuracy of judgments by influencing processes that operate on the products of retrieval, such as post-retrieval monitoring processes. Some recent data from our lab (Herron, 2018) indicates that there is in fact a trade-off between the maintenance of retrieval orientations and post-retrieval monitoring. In this study, participants were required to complete two blocked memory tasks with different retrieval goals. Half the participants completed a stroop task prior to testing, with the intention of depleting resources of cognitive control and thereby reducing their opportunity to engage and maintain task-appropriate retrieval orientations, while the other half read color names printed in black ink. While the control group showed a robust pre-stimulus ERP effect consistent with the maintenance of different retrieval orientations throughout the two tasks, this effect was absent in the stroop group. Conversely, a right frontal stimulus-locked old/new effect (1100–1400ms) strongly associated with post-retrieval monitoring (Friedman and Johnson, 2000, Mecklinger, 2000, Wilding and Ranganath, 2011) was observed only for the stroop group, with retrieval accuracy being equivalent across the two groups. The degree to which retrieval orientations are initiated and maintained therefore appears to influence retrieval efficiency by reducing the need for compensatory post-retrieval monitoring processes.

Finally, retrieval orientations may also influence retrieval accuracy by priming brain regions required for episodic memory prior to encountering a test item (Morris et al., 1977, Polyn and Kahana, 2008), in much the same way as has been demonstrated in studies of perception (Chawla, Rees, & Friston, 1999). Using event-related fMRI, Chawla et al. (1999) found that selective attention to either color or movement attributes modulated between-stimulus baseline activity in color- or motion-sensitive areas of extrastriate cortex (areas V4 and V5 respectively), with visually-evoked responses to task-relevant stimuli increasing alongside these baseline enhancements. Attentional orienting to the contents of memory may operate in a similar way, and Leynes and colleagues (Bruett and Leynes, 2015, Leynes and Zish, 2012) have proposed that top-down fluency-sensitive processes operating across test items can allow fluency to support memory judgments. For example, it has been shown that manipulating the visual clarity of memory probes (Leynes & Zish, 2012) results in elevated levels of fluency being attributed to the encoding phase. Importantly for the present findings, these fluency-related ERP memory effects were evident when stimulus fluency was varied randomly throughout the memory test but not when it was blocked, indicating that the trial sequence was important in obtaining these findings. To the extent that fluency can support source judgments (Leynes, Askin, & Landau, 2017), it is possible that these fluency-sensitive top-down processes could be reflected in the preparatory ERPs reported here. Further studies using different memory tasks will reveal whether these preparatory differences vary qualitatively according to contextual retrieval requirements as would be predicted for correlates of retrieval orientations.

If we are correct in our assertion that mode should predict all forms of episodic memory, the absence of any effect between Episodic Hit-Misses and Non-Episodic Hits indicates that this contrast did not capture the correlate of retrieval mode. This is somewhat surprising given the fact that preparatory correlates of mode initiation have been reported in both recognition (Duzel et al., 2001, Duzel et al., 1999, Morcom and Rugg, 2002, Wilckens et al., 2011) and source memory tasks (Evans et al., 2015, Herron and Wilding, 2004, Herron and Wilding, 2006a). While none of these experiments were able to separate preparatory neural activity according to subsequent memory accuracy – and therefore reported general effects of cue-type only – the high levels of memory accuracy in these studies indicate that these effects predominantly preceded memory success in the tasks under investigation. There are at least two possibilities for the absence of the index of retrieval mode in the current study: i) participants engaged only in retrieval orientation and not mode, or ii) participants failed to disengage from mode during the smaller number of non-episodic trials and hence the right frontal effect that has been linked to the initiation of retrieval mode would not be observed. The first interpretation would be problematic for the theoretical concept of mode, as this is considered to be initiated whenever episodic retrieval is required. We do not believe that the literature supports this interpretation of our data, as right-lateralised preparatory correlates of retrieval mode have been reported both for the same source memory task that we employed here (Herron & Wilding, 2004) and for a similar source memory task requiring the retrieval of location-based information (Evans et al., 2015, Herron and Wilding, 2006a). But if the second interpretation is correct, why would participants fail to disengage from mode during the non-episodic task? There were a number of asymmetries between the episodic and non-episodic task, necessary in order to obtain sufficient numbers of memory errors within a recording session of reasonable length while retaining memory performance that was clearly above chance. These included the higher proportion of episodic to non-episodic cues (2:1, excluding filler trials), the higher proportion of studied to unstudied items (3:1), and the greater relative difficulty of the episodic task. While Herron and Wilding (2004) obtained neural correlates of mode with a 2:1 ratio of episodic to non-episodic cues, the other two factors could have predisposed participants to remain in mode. For example, it has been demonstrated in the task-switching literature that carryover effects are influenced by relative task difficulty, with more difficult tasks that require a greater degree of cognitive control having greater carryover effects into the alternate task (Allport et al., 1994, Monsell et al., 2000, Yeung and Monsell, 2003).

Finally, it is noteworthy that Addante, Watrous, Yonelinas, Ekstrom, and Ranganath (2011) have also reported neural activity that predicted the accuracy of source memory judgments. In contrast to the current study there was no switching requirement, participants only completed an episodic task, and the effect was in the time-frequency domain. These researchers found that frontal/temporal theta activity in the 300 ms prior to stimulus presentation predicted accurate retrieval of contextual information. The transient nature of this oscillatory effect is inconsistent with that expected of a memory state, so it is likely that their data do not speak to the same theoretical questions as ours. It is also difficult to determine which cognitive processes are reflected in the oscillatory index due to the lack of other tasks (e.g. episodic or non-episodic) to compare it to and the paucity of other oscillatory studies in this area. Nonetheless, their findings suggest the existence of more temporally constrained pre-retrieval processes, in addition to the sustained state-related effects reported here, and these might well exert their influence on subsequent retrieval processing operations in different ways.

In conclusion, this is the first study to demonstrate directly that the initiation of an episodic memory state predicts the accuracy of source memory judgments. This frontally distributed effect was evident on switch trials, was not right-lateralised, and further dissociated Episodic Hit-Hits from Episodic Hit-Misses (thereby indicating a specific role in criterial recollection), which all point to an index of retrieval orientation as opposed to retrieval mode. There are significant opportunities to pursue further research which will permit specification of the cognitive and neural processes that support memory states, as well as a characterization of the retrieval processes that they promote.

## Author contributions

L. H. Evans developed the study concept. Both authors contributed to the study design. Data collection and statistical analyses were performed by J.E. Herron. Both authors drafted the manuscript and approved the final version for publication.

## Declaration of conflicting interests

The authors declared that they had no conflicts of interest with respect to their authorship or the publication of this article.

## Funding

This research was funded by the Bial Foundation (91/12) to Lisa Evans. Jane Herron is currently supported by the Wellcome Trust (106278/Z/14/Z).

## Footnotes

1 There were insufficient trials associated with Episodic Miss-Misses responses (i.e. studied items which were not recognized at all) to form reliable averaged ERPs for this response type.
